# Long-Term Shape and Volume Retention of Acellular Dermal Matrix in Oncoplastic Breast-Conserving Surgery: A 2-Year Retrospective Study

**DOI:** 10.3390/jcm14093002

**Published:** 2025-04-26

**Authors:** Hyung-suk Yi, Jeong-jin Park, Jin-hyung Park, Hong-Il Kim, Jong-Hyouk Yun, Sung-ui Jung, Jin-hyuk Choi, Ku-sang Kim, Yoon-soo Kim

**Affiliations:** 1 Department of Plastic and Reconstructive Surgery, Kosin University Gospel Hospital, Kosin University College of Medicine, Busan 49267, Republic of Korea; sencha21@naver.com (H.-s.Y.); jayjay03157@gmail.com (J.-j.P.); atreyue@naver.com (J.-h.P.); immtkg4u@hanmail.net (H.-I.K.); 2 Department of Radiology, Kosin University Gospel Hospital, Kosin University College of Medicine, Busan 49267, Republic of Korea; xell1015@naver.com; 3 Department of Surgery, Kosin University Gospel Hospital, Kosin University College of Medicine, Busan 49267, Republic of Korea; ist2000good@daum.net (S.-u.J.); drchoijinhyuk@gmail.com (J.-h.C.); ideakims@gmail.com (K.-s.K.)

**Keywords:** volume stability, MRI volumetric analysis, breast reconstruction, radiotherapy outcomes, tissue integration, diced ADM, paste ADM, sheet ADM, breast conservation techniques, quantitative assessment

## Abstract

**Background/Objectives**: To quantitatively assess the long-term volume stability of acellular dermal matrix (ADM) in oncoplastic breast-conserving surgery (OBCS) and analyze surgical and aesthetic outcomes. **Methods**: This retrospective study examined 172 breast cancer patients who underwent OBCS with immediate ADM-based volume replacement (2020–2022). Patients received either diced ADM with sheet ADM (*n* = 102) or diced ADM with paste ADM (n = 70). The ADM volume was evaluated using MRI at 6, 12, and 24 months postoperatively. **Results**: Long-term volume stability was achieved in both groups with minimal volume reduction (4.3–4.5%) at 24 months (*p* < 0.001). Early surgical complications included hematoma (4.1%), seroma (2.3%), and wound issues (1.2%), with no infections or ADM non-incorporation. Contour irregularities occurred in 16.3% of cases. Radiotherapy (87% of patients) did not significantly impact ADM volume retention. **Conclusions**: ADM provides predictable, durable volume replacement in OBCS, with excellent volume stability even with radiotherapy. This quantitative assessment of ADM volume retention over two years supports ADM as a reliable option for breast-conserving surgery, potentially expanding treatment options for patients with unfavorable tumor-to-breast volume ratios.

## 1. Introduction

Oncoplastic breast-conserving surgery (OBCS) has revolutionized the treatment of early-stage breast cancer by offering oncological outcomes comparable to mastectomy while preserving the breast’s natural appearance [1,2]. However, significant volume loss and breast shape distortion following large tumor resections remain challenging aspects of traditional breast-conserving surgery (BCS), particularly when the tumor-to-breast volume ratio is high [3].

Although OBCS has been proven oncologically safe, careful consideration must be given to tumor size and location when preserving breast tissue [4,5]. The volume of tissue removed in oncoplastic breast-conserving surgery depends on multiple factors, including the patient’s BMI, breast size, and tumor size; however, the precise amount of tissue removal is often difficult to predict preoperatively, making reconstruction challenging and potentially requiring different surgical approaches based on tumor location [6,7].

The integration of acellular dermal matrix (ADM) for volume replacement in OBCS has emerged as a promising solution to address these challenges [8,9]. As plastic surgeons continue seeking ways to restore post-cancer defects, various techniques have been developed using ADM with round block approaches or autologous tissue [10,11]. ADM provides a biocompatible scaffold for breast reconstruction [12]. The removal of cellular components reduces immunogenicity while maintaining the structural integrity of the matrix [13]. Furthermore, ADM promotes angiogenesis and cellular repopulation, facilitating integration with surrounding breast tissue [14]. These characteristics make ADM particularly suitable for volume replacement in OBCS, allowing immediate filling of defects and potentially improving breast contour and symmetry [15,16].

Despite the increasing adoption of ADM in OBCS, several critical questions remain unanswered. While short-term outcomes appear promising, the long-term stability of the ADM volume remains poorly understood, particularly beyond the first postoperative year [17,18]. Additionally, the potential impact of adjuvant radiotherapy on ADM volume retention has not been thoroughly investigated [19]. Furthermore, there are currently no clear guidelines regarding the optimal ratio of ADM volume to excised breast tissue, nor is there consensus on the most effective method of ADM preparation and placement.

Different ADM preparation techniques have been developed to optimize outcomes, including the combination of diced ADM with either sheet ADM or paste ADM. Each combination offers distinct advantages, but comparative data on their long-term performance are limited. The sheet ADM overlay may provide additional support and prevent contour irregularities, while paste ADM offers superior malleability for filling irregular defects. However, the relative effectiveness of these approaches in maintaining long-term volume and aesthetic outcomes remains unclear.

To address these knowledge gaps, we conducted a retrospective review of 172 patients who underwent OBCS with ADM reconstruction at our institution. We hypothesized that ADM would provide durable volume replacement with minimal degradation over time, regardless of adjuvant radiotherapy administration. Using serial MRI measurements over a two-year period, we quantitatively assessed changes in the ADM volume and analyzed factors that might influence volume retention. Our study specifically aimed to (1) quantitatively evaluate the long-term volume stability of ADM in OBCS, (2) compare outcomes between different ADM combinations (diced with sheet versus diced with paste), and (3) assess the impact of radiotherapy on volume maintenance.

## 2. Materials and Methods

### 2.1. Patient Selection

This retrospective study included 172 breast cancer patients who underwent oncoplastic breast-conserving surgery (BCS) with immediate volume replacement using acellular dermal matrix (ADM) between May 2020 and May 2022. Patients were divided into two groups based on the ADM combination used: diced ADM combined with sheet ADM (n = 102) and diced ADM combined with paste ADM (n = 70).

The inclusion criteria were as follows: (1) patients with histologically confirmed breast cancer, (2) patients who received preoperative counseling regarding potential complications and depression deformity following BCS, (3) patients who consented to immediate breast reconstruction using ADM for volume replacement, and (4) patients who completed postoperative MRI follow-up at 6, 12, and 24 months.

Exclusion criteria included the following: (1) patients with a history of previous breast surgery, (2) patients with collagen vascular disease or other connective tissue disorders, (3) patients who could not undergo MRI examination due to contraindications, and (4) patients who developed local recurrence during the follow-up period.

ADM was utilized regardless of defect location and shape. Two plastic surgeons (JHP and YSK) performed the immediate ADM-based reconstructions following the completion of partial mastectomy and axillary staging (sentinel lymph node biopsy or axillary lymph node dissection, as indicated per standard guidelines) conducted by the breast surgeons.

Demographic and clinical data were extracted from patients’ medical records, including medical history, tumor characteristics (type and location), resected breast tissue weight and volume, ADM volume used, and details of neoadjuvant/adjuvant chemotherapy and radiation therapy.

The study protocol was approved by the Institutional Review Board of Kosin University Gospel Hospital (approval number KUGH 2024-08-016, approval date 14 August 2024). All participants provided informed consent for their medical information to be stored in the database and used for research purposes. The study was conducted in accordance with the principles of the 1964 Declaration of Helsinki and its later amendments.

### 2.2. MRI Protocol and Volumetric Analysis

Follow-up MRI examinations were performed at 6, 12, and 24 months postoperatively to assess ADM volumetric changes and for cancer surveillance. All MRIs were performed using a 3T breast MRI system (Discovery MR 750w, General Electric Healthcare, Waukesha, WI, USA).

The imaging protocol included:

- Axial, sagittal, and coronal turbo spin-echo T2-weighted imaging (T2WI).
- Echo-planar diffusion-weighted imaging (DWI).
- Spoiled gradient echo dynamic contrast-enhanced (DCE) sequences.
- Sagittal T2WI: 5 mm slice thickness (4 mm with 1 mm gap).
- Axial T2WI and T1-weighted imaging (T1WI): 7 mm slice thickness (5 mm with 2 mm gap).

Two sets of T2WI were acquired using conventional (4 min) and short (2 min 30 s) acquisition times, with a matrix size of 512 × 512 and a field of view of 180 × 180 mm. All MRI examinations were performed with patients in the prone position using a dedicated breast coil to minimize motion artifacts and optimize breast tissue visualization, maintaining consistent positioning across all follow-up time points to ensure reliable volumetric comparisons.

### 2.3. Volumetric Analysis of ADM

**Figure 1:**
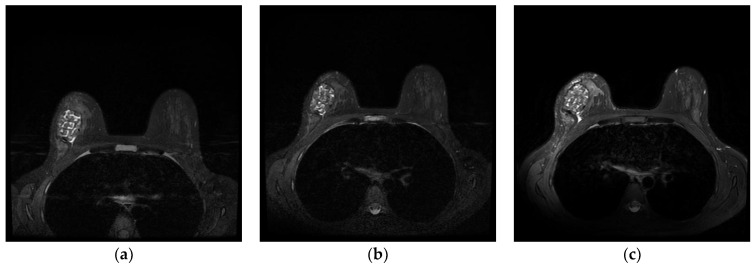
Serial magnetic resonance imaging demonstrating long-term volume stability of diced ADM with paste-type ADM in a 40-year-old patient who underwent oncoplastic breast-conserving surgery. T2-weighted axial images show consistent low signal intensity of the ADM construct at (**a**) 6 months postoperatively, (**b**) 12 months postoperatively, and (**c**) 24 months postoperatively. Quantitative analysis revealed minimal volume reduction (3.7%) over the 24-month follow-up period, confirming the long-term stability of the ADM reconstruction. Note the well-defined margins and homogeneous signal characteristics of the ADM throughout the follow-up period, indicating successful tissue integration without significant degradation or resorption.

Quantitative analyses of the ADM volumes were performed at three time points postoperatively: 6, 12, and 24 months. The MRI data were assessed by three reviewers: one radiologist specializing in breast MRI (JHY) and two plastic surgeons experienced in oncoplastic breast reconstruction. The reviewers independently evaluated all serial MRI studies for each of the 172 patients. Areas exhibiting moderate to low signal intensity on T2-weighted images that corresponded to the location of the ADM were identified (Figure 1).

Two methods were used to calculate the ADM volume at each time point: the conventional diameter-based method and a semi-automated regions of interest (ROI)-based 3D volumetry technique.

For the diameter-based approach, the maximum dimensions of the ADM were measured in three orthogonal planes: craniocaudal (d~cc~) and anteroposterior (d~ap~) diameters on sagittal images, and lateral diameter (d~l~) on axial images. ADM volume was then calculated using the formula for an ellipsoid: (V = d~cc~ × d~ap~ × d~l~ × π/6)

For the ROI-based 3D volumetry, the entire ADM region was manually delineated on each slice of the sagittal T2-weighted images using ImageJ software (version 1.53, National Institutes of Health, USA). The semi-automated tool in ImageJ was then used to generate a 3D volumetric reconstruction of the ADM, with the final volume calculated by summing the areas of each ROI and multiplying by the slice thickness.

The ROI-based method offers several advantages over the diameter-based method: (1) it accounts for irregular shapes that deviate from perfect ellipsoids, (2) it provides more accurate measurements of complex three-dimensional structures, (3) it reduces observer-dependent variations in measurement, and (4) it allows for better visualization and quantification of volume changes in specific regions within the ADM.

ADM density was also assessed qualitatively on the T2-weighted images and classified as homogeneous or heterogeneous. The presence of any areas of possible fluid collection, fat necrosis, or residual breast tissue within the ADM was noted.

### 2.4. Operation Procedure

The surgical approach was standardized across all cases. Preoperatively, incision design was marked by a plastic surgeon with consideration of tumor size and nipple–tumor distance. A peri-areolar or direct incision was planned, overlying the tumor site, confirmed by ultrasound guidance.

Following the predetermined design, the breast surgeon performed partial mastectomy with sentinel lymph node biopsy. Tumor extirpation inherently creates a surgical plane that extends beyond the primary defect, as dissection between the breast parenchyma and pectoralis major muscle is necessary to ensure adequate oncologic margins. This dissection results in a potential space significantly larger than the actual tumor defect, which, if left unaddressed, could allow unwanted migration of the ADM and compromise reconstruction outcomes. To optimize the reconstructive environment, we employed a systematic approach to pocket preparation before ADM placement. The elevated glandular flaps were methodically reapproximated to the pectoralis major muscle using interrupted 3-0 Vicryl sutures (Ethicon Inc., Somerville, NJ, USA). This critical maneuver serves multiple purposes: it eliminates potential dead space, prevents ADM migration into unwanted planes, reduces the risk of seroma formation, and most importantly, creates a well-defined cavity that precisely corresponds to the volume of resected tumor tissue. This controlled pocket preparation ensures that the ADM replacement volume matches the actual tissue defect rather than the larger surgical field created during oncologic resection. After securing the breast parenchyma and confirming adequate hemostasis, a Jackson–Pratt (JP) drain was positioned within the tumor cavity to manage postoperative fluid collection.

**Figure 2:**
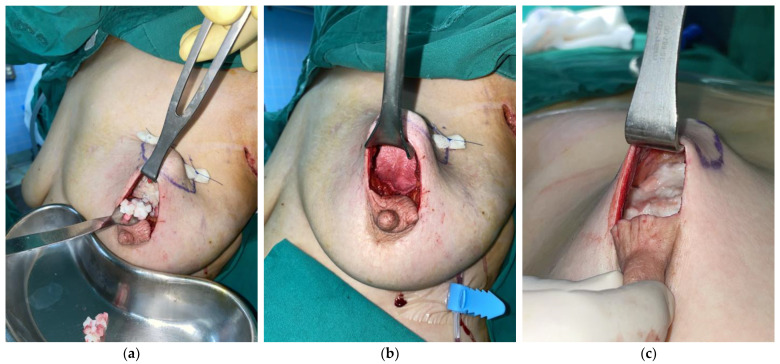
Intraoperative photographs demonstrating different ADM application techniques. (**a**,**b**) Diced and sheet ADM combination technique in the same patient: (**a**) Following resection of 33 g of breast tissue from the upper outer quadrant of the left breast, 29 cm^3^ of diced ADM has been placed into the defect cavity. (**b**) Subsequently, 8 cm^3^ of sheet ADM is positioned beneath the skin flap to provide additional support and prevent contour irregularities (total ADM volume utilized: 37 cm^3^, achieving a replacement ratio of 1.12). (**c**) Alternative technique in a different patient: after excision of 16 g of breast tissue, approximately 18 cm^3^ of a mixture of diced ADM and paste ADM was inserted (replacement ratio: 1.13).

The volume of ADM used for reconstruction was calculated based on the weight of resected breast tissue, with a replacement ratio of 1.1 to 1.2 times the resected weight. In cases with thin overlying skin flaps or visible contour irregularities using diced ADM alone, diced ADM was inserted under the skin flap with additional sheet-type ADM placed beneath the skin flap for reinforcement. The sheet ADM was secured using 3-0 Vicryl sutures and a pullout technique with 2-0 Prolene sutures (Ethicon Inc.) and bolsters. For defects distant from the skin flap or with adequate flap thickness, a combination of paste and diced ADM was inserted, providing optimal filling of irregular defects while maintaining volume stability (Figure 2).

The study utilized Megaderm Dice^®^ (L&C Bio, Seoul, Korea), a cross-linked human ADM measuring 3–5 mm in thickness and available in 1 × 13 cm or 2 × 11 cm dimensions for diced applications. Paste ADM consisted of Megafill^®^ (L&C Bio), comprising micronized ADM particles mixed with saline in ratios of 1 cc or 2.5 cc ADM with 3.8 cc and 9.5 cc saline, respectively. Sheet ADM applications employed CGCryoDerm^®^ (CGBIO Inc., Seongnam, Korea), a cross-linked human ADM of 2–3 mm thickness in 3 × 4 cm dimensions. All diced- and sheet-type ADMs underwent presoaking in povidone–iodine solution followed by saline irrigation for 5 min intraoperatively.

After ADM placement, patients were positioned upright to assess for contour depression. Final closure was performed in layers using 4-0 PDS for dermal closure and 5-0 Nylon sutures for skin closure.

### 2.5. Postoperative Management

Postoperative molding, when required, was performed during dressing changes. DuoDERM CGF^®^ (Convatec Inc., Gangnam, Korea) was applied around the ADM insertion site to prevent compression-related contour depressions. The JP drains were removed when the output was less than 10 mL/day, which occurred at an average of 5 days postoperatively. Intravenous ceftazidime (2 g/day) was administered until 48 h after surgery.

Aesthetic outcomes were evaluated by a panel of 3 independent observers (2 plastic surgeons not involved in the surgeries and 1 breast surgeon) at 12 and 24 months postoperatively. The assessment utilized a standardized four-point scale (0 = poor, 1 = fair, 2 = good, and 3 = excellent) evaluating breast symmetry, contour, and overall aesthetic outcome. The evaluators were blinded to the type of ADM used.

### 2.6. Statistical Analysis

All statistical analyses were performed using SPSS software (version 18.0, SPSS Inc., Chicago, IL, USA). Statistical significance was defined as *p* < 0.05. Descriptive statistics were used to summarize patient demographics, tumor characteristics, and surgical details. Continuous variables were expressed as mean ± standard deviation (SD), and categorical variables as counts and percentages.

ADM volumes derived from both diameter-based and ROI-based methods were compared at each of the three postoperative time points using repeated measures analysis of variance (ANOVA). Pairwise comparisons were performed with Bonferroni correction for multiple testing.

To evaluate factors influencing ADM volume changes over time, subgroup analyses were conducted to assess the impact of radiation therapy and ADM type. Multivariate linear regression analysis was performed to identify independent predictors of ADM volume change over time, with variables including age, BMI, tumor location, radiation therapy, and ADM type.

Between-group comparisons for categorical variables, like complication rates, were performed using Chi-square or Fisher’s exact tests, while continuous variables, like aesthetic scores, were compared using independent t-tests or Mann–Whitney U tests, as appropriate, based on data distribution.

Early surgical complications and aesthetic outcomes were analyzed separately to provide clear differentiation between technical and cosmetic results. The relationship between preoperative factors and surgical outcomes was evaluated using appropriate statistical tests: chi-square or Fisher’s exact test for categorical variables, and independent t-test or Mann–Whitney test for continuous variables based on normality of distribution.

Sample size calculation was based on previously published data on ADM volume retention in breast reconstruction. With an anticipated difference in volume retention of 10% between groups, a standard deviation of 15%, an alpha level of 0.05, and a power of 80%, we calculated that a minimum of 160 patients would be required. Our sample of 172 patients exceeded this requirement.

## 3. Results

### 3.1. Patient Characteristics

The study included 172 breast cancer patients who underwent oncoplastic breast-conserving surgery with either diced ADM combined with sheet ADM (n = 102), or diced ADM combined with paste ADM (n = 70). The mean age was 49.3 ± 9.2 years (range, 25–73 years) and mean BMI was 23.7 ± 3.2 kg/m^2^ (range, 18–34.5 kg/m^2^). Tumor locations were predominantly in the superolateral (42%) and superomedial (28%) quadrants. Radiotherapy and chemotherapy were administered in 98.7% and 75% of patients, respectively. The most common histologic type was invasive ductal carcinoma (72%), followed by ductal carcinoma in situ (22%).

The mean resected tissue weight was 21.5 ± 8.8 g (range, 5–48 g), with corresponding volumes of 20.9 ± 8.8 cm^3^ (range, 5.3–48.1 cm^3^). The mean ADM volume utilized was 23.7 ± 6.2 cm^3^ (range, 7.7–36 cm^3^), maintaining the planned 1.1 to 1.2 replacement ratio (Table 1).

### 3.2. Surgical Complications

Early surgical complications occurred in 7.6% of cases (13 patients), including hematoma (4.1%), seroma (2.3%), and wound healing issues (1.2%). No cases of infection, flap necrosis, or ADM non-incorporation were observed during the follow-up period. The complication rates were comparable between ADM combinations, demonstrating similar safety profiles (Table 2).

### 3.3. Volume Retention Analysis

**Figure 3:**
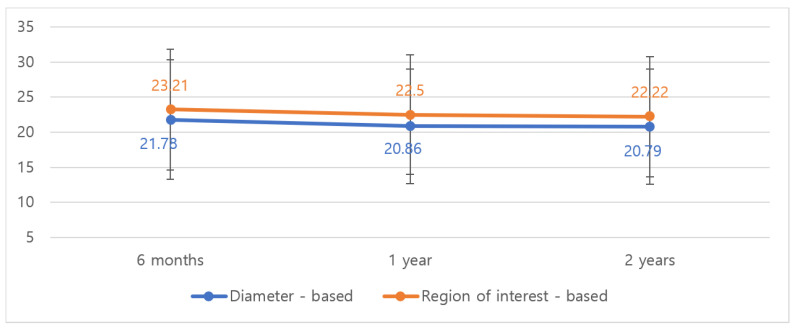
Volumetric change of diced- and sheet-type ADM on MRI follow-up at 6 months, 1 year, and 2 years. Both diameter-based and ROI-based measurements showed minimal volume reduction (4.5% and 4.3%, respectively) over the 2-year follow-up period.

Serial MRI evaluation revealed consistent volume stability in both groups (Figure 3). The diced ADM with sheet ADM group showed a volume decrease from 21.78 cm^3^ to 20.79 cm^3^ by diameter-based measurement and from 23.21 cm^3^ to 22.22 cm^3^ by ROI-based measurement over 2 years (*p* < 0.001; Table 3). This represented a modest 4.5% and 4.3% decrease in volume by diameter-based and ROI-based methods, respectively.

In the diced ADM with paste ADM group, the median volume decreased from 18.35 cm^3^ to 17.80 cm^3^ by diameter-based measurement and from 19.53 cm^3^ to 18.81 cm^3^ by ROI-based measurement over 2 years (*p* < 0.001; Table 3). The volume reduction patterns were similar between groups, with most changes occurring within the first six months postoperatively.

**Figure 4:**
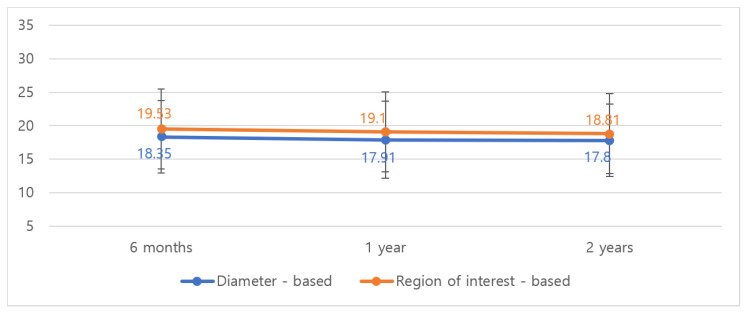
Volumetric changes of diced- and paste-type ADM on MRI follow-up at 6 months, 1 year, and 2 years. Similar to the sheet ADM group, volume stabilization was observed after the initial 6 months, with a total volume reduction of only 3.7% over 24 months.

Figure 4 illustrates the volume changes over time for both ADM combinations, showing the initial slight decrease followed by stabilization after 12 months.

### 3.4. Impact of Radiotherapy

**Figure 5:**
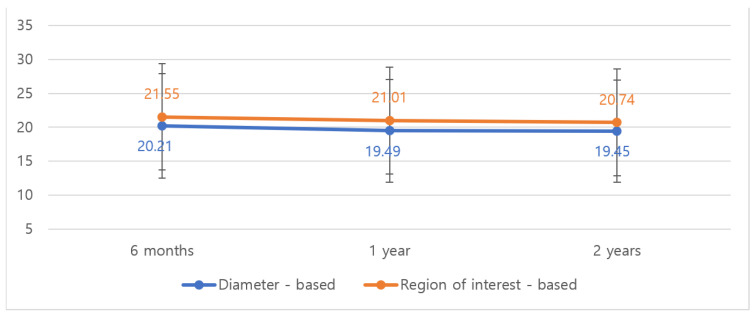
Volumetric changes in ADM at MRI follow-up at 6 months, 1 year, and 2 years in patients who received radiotherapy (n = 149). Radiotherapy did not significantly impact the rate or extent of volume loss, with only a 3.8% total volume reduction observed at 24 months.

Among patients receiving radiotherapy (98.7%), the median volume decreased from 20.21 cm^3^ to 19.45 cm^3^ by diameter-based measurement and from 21.55 cm^3^ to 20.74 cm^3^ by ROI-based measurement over 2 years (*p* < 0.001; Figure 5 and Table 3). Notably, radiotherapy did not significantly impact the rate or extent of volume loss in the vast majority of cases. Multivariate analysis confirmed that radiation therapy was not an independent predictor of ADM volume reduction (*p* = 0.38).

### 3.5. Aesthetic Assessment

Contour irregularities requiring revision occurred in 16.3% of cases, including bulging deformity (9.9%) and depression deformity (6.4%; Table 4). These aesthetic concerns were successfully addressed through minor revision procedures. Both ADM combinations demonstrated comparable rates of aesthetic refinement needs.

The mean aesthetic scores at 24 months postoperatively were 2.4 ± 0.6 for the diced with sheet ADM group and 2.3 ± 0.7 for the diced with paste ADM group, showing no statistically significant difference between the two techniques (*p* = 0.52).

**Figure 6:**
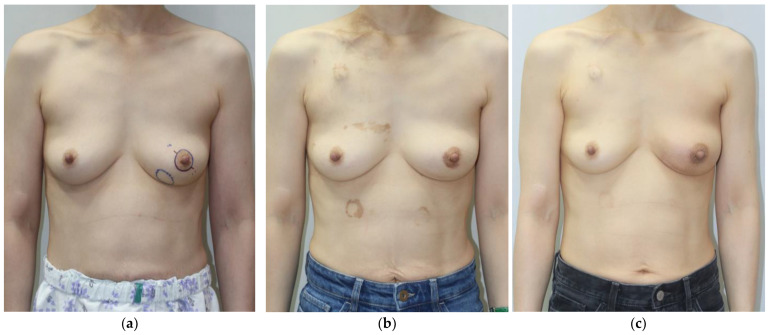

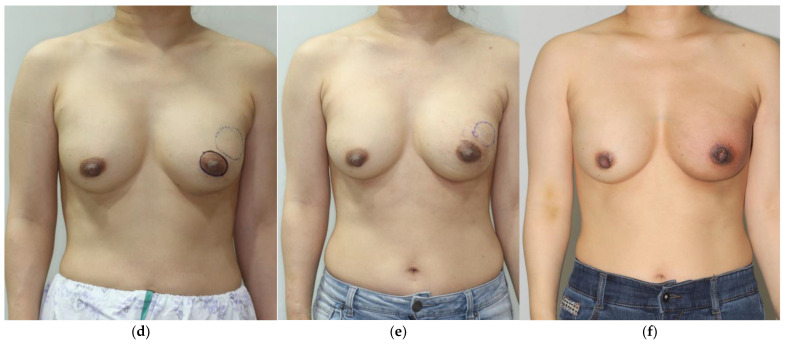
Preoperative (**a**,**d**), postoperative 6-month (**b**,**e**), and postoperative 12-month (**c**,**f**) photographs. (**a**–**c**) A 45-year-old patient’s tumor was located in the lower inner quadrant of the left breast, and the resected breast tissue was 21 g. In total, 24.5 cm^3^ of diced ADM and paste ADM was used. (**d**–**f**) A 40-year-old patient’s tumor was located in the upper outer quadrant of the left breast, and the resected breast tissue was 29.5 g. In total, 33 cm^3^ of diced ADM and sheet ADM was used.

Representative pre- and post-operative photographs are shown in Figure 6, demonstrating the natural contour and volume maintenance achieved with both ADM combinations.

## 4. Discussion

This study represents one of the first long-term quantitative assessments of ADM volume retention in oncoplastic breast-conserving surgery, demonstrating remarkable stability with only 4.3–4.5% volume reduction at two years. This finding has significant implications for expanding breast conservation options to patients who would traditionally require mastectomy due to unfavorable tumor-to-breast volume ratios [20,21]. The stability we observed, combined with the low complication rate, suggests that ADM-based volume replacement should be considered a standard option in the oncoplastic surgery armamentarium.

Perhaps most importantly, our finding that radiotherapy did not significantly impact ADM volume retention—even with 98.7% of patients receiving radiation—challenges previous concerns about combining ADM reconstruction with radiation therapy [22,23]. This observation is particularly relevant for clinical decision-making, as it suggests that ADM-based reconstruction can be safely offered to patients requiring postoperative radiation, expanding treatment options for this challenging patient population. These results align with recent findings by Chatterjee et al. [24] and Lee et al. [25], who reported minimal impact of radiation on ADM stability in implant-based reconstruction, though our study extended these observations to partial breast reconstruction scenarios.

Our findings of minimal volume reduction (4.3–4.5%) over 24 months align with recent work by Kim et al. [26], who reported similar stability using CT volumetry following ADM-based volume replacement. Their work demonstrated comparable volume retention rates despite different imaging modalities and ADM preparation techniques, suggesting the inherent biological properties of ADM may be more significant than technical variations in preparation.

Our observation that radiotherapy did not significantly impact ADM volume retention aligns with emerging evidence that ADM may confer protective effects in irradiated settings. Azzena et al. [27] recently demonstrated reduced capsular contracture rates in ADM-assisted prepectoral reconstructions exposed to radiotherapy compared to non-ADM reconstructions. Together, these findings suggest that ADM may possess unique biological properties that enhance radiation resistance across various breast reconstruction applications.

Importantly, the volume stability we observed contrasts significantly with expected tissue changes following conventional breast-conserving therapy. Mennie et al. [28] recently documented five-year longitudinal volume changes showing 12–18% volume loss in irradiated breasts following standard lumpectomy without volume replacement. The marked difference between these findings and our observed 4.3–4.5% reduction highlights the potential value of ADM in mitigating post-radiotherapy volume loss.

Our comparison of diced ADM combined with either sheet ADM or paste ADM provides practical guidance for surgical technique selection. Both combinations demonstrated comparable safety profiles and volume stability, suggesting that surgeons can choose between these approaches based on specific defect characteristics and tissue requirements rather than concerns about differential volume retention [29]. The versatility of these techniques allows surgeons to adapt their approach to various tumor locations and defect volumes while maintaining consistent outcomes.

The choice between sheet- and paste-type ADM supplementation may be further refined based on tumor location, as demonstrated in recent work by Yi et al. [30]. Their analysis suggested that specific quadrants of the breast may benefit differentially from various ADM preparation techniques, with medial quadrant defects potentially requiring additional structural support compared to lateral defects. This anatomical consideration warrants further investigation in prospective studies.

The relationship between radiotherapy and reconstructed tissue volume remains complex across various reconstructive modalities. Fischer et al. [31] documented variable volume changes (4–22%) in autologous tissue following radiation exposure. The relatively modest volume reduction observed in our ADM cohort suggests potential advantages over certain autologous options in the setting of adjuvant radiotherapy, though direct comparative studies are needed to confirm this observation.

The technical aspects of our approach warrant specific discussion, as they likely contributed to the favorable outcomes observed. Our systematic method of pocket preparation, which included careful re-approximation of glandular flaps to the pectoralis major muscle, created a well-defined cavity that precisely corresponded to the resected tissue volume. This controlled pocket preparation served multiple purposes: it eliminated potential dead space, prevented unwanted ADM migration, reduced seroma formation, and ensured the ADM replacement volume matched the actual tissue defect rather than the larger surgical field created during oncologic resection. We believe this attention to technical detail contributed significantly to the long-term volume stability observed in our series, consistent with principles emphasized in recent publications by Kim et al. [26] and Azzena et al. [27].

ADM-based volume replacement represents one of several available reconstructive strategies for oncoplastic breast-conserving surgery. Alternative approaches include autologous fat grafting, which offers natural consistency but may be limited by unpredictable volume retention and potential screening interference, local tissue rearrangement techniques, such as thoracoepigastric or lateral thoracic flaps, which provide robust vascularized tissue but entail additional donor site morbidity, and synthetic mesh implantation, which offers structural support but with potentially higher rates of extrusion and infection compared to ADM. Our findings suggested that ADM may occupy a valuable middle ground, providing predictable volume stability without donor site morbidity, while demonstrating particular resilience to radiotherapy—a significant advantage over certain autologous options that may exhibit greater volume variability following radiation.

The early surgical complication rate in our study (7.6%) compared favorably with previously reported rates for oncoplastic breast surgery, which ranged from 5.1% to 25.9% [28,32]. When complications did occur, they were predominantly manageable issues, such as hematoma (4.1%) and seroma (2.3%), that resolved with standard interventions. This safety profile supports the broader adoption of ADM-based volume replacement techniques in appropriate candidates.

The mean operative time was comparable between techniques (diced ADM with sheet ADM: 78.4 ± 12.5 min; diced ADM with paste ADM: 75.2 ± 11.8 min; *p* = 0.38), suggesting that the technical differences did not significantly impact surgical duration. Similarly, the mean length of hospitalization was consistent across all groups (6.2 ± 0.3 days). Drain removal occurred at a mean of 5.3 ± 1.7 days postoperatively, with no significant differences between ADM techniques (*p* = 0.76). At our institution, patients typically remained hospitalized until drain removal to ensure optimal wound management and minimize infection risk under direct clinical supervision. These parameters indicate that ADM-based volume replacement can be efficiently integrated into the surgical workflow without extending the operative time or hospitalization beyond what is typically required for standard breast-conserving procedures at our center.

Alternative ADM preparation techniques continue to evolve, with recent case reports by Lee et al. [33] demonstrating promising results using novel 3D grid and strip-shaped configurations. These emerging approaches may further expand the armamentarium of ADM-based volume replacement techniques, potentially offering additional options for specific defect morphologies while maintaining the volume stability demonstrated in our series.

Our approach offered several distinct advantages over traditional volume displacement techniques. First, it provided precise volume replacement, addressing a common limitation of conventional breast conservation therapy where volume discrepancies can lead to asymmetry [30]. Second, the stability of volume retention may reduce the need for contralateral symmetrization procedures, potentially decreasing the overall surgical burden for patients [34]. Third, the technique’s versatility permitted reconstruction of defects throughout the breast while maintaining natural contour and ptosis [31].

The aesthetic outcomes in our series deserve particular attention. While contour irregularities occurred in 16.3% of cases, most were successfully addressed through minor revisions. The higher rate of bulging deformity (9.9%) compared to depression deformity (6.4%) suggested that slight under-correction may be preferable when determining the ADM volume. This observation has influenced our current practice, where we now tend to use replacement volumes at the lower end of our calculated range. Similar adaptive strategies have been reported by Urban et al. [35] and Holmes et al. [36] in their series on oncoplastic reconstruction techniques. Based on our observation that bulging deformity (9.9%) occurred more frequently than depression deformity (6.4%), we refined our intraoperative protocol for ADM volume determination. Our current practice employs a more conservative approach, targeting a replacement ratio of 1.0–1.1 times the resected weight (rather than the previous 1.1–1.2 ratio) for most cases, with particular attention to avoiding overcorrection in the upper breast quadrants, where even minor excess volume can create noticeable asymmetry. Additionally, we now implement systematic intraoperative patient positioning at 30°, 60°, and 90° upright to assess dynamic contour changes before final closure, allowing for precise volume adjustments.

The ROI-based volumetric method used in our study represents an advancement over traditional diameter-based measurements. By accounting for the three-dimensional shape of the ADM, this technique provides more accurate volume calculations, especially for irregularly shaped reconstructions [37]. The consistent findings between both measurement methods enhanced the reliability of our results and suggested that either approach can be used in clinical practice, though ROI-based techniques may offer advantages for research applications requiring precise quantification.

Our findings must be interpreted in the context of certain limitations. The retrospective design introduces potential selection bias, although our consecutive case series helps mitigate this concern. Second, while our two-year follow-up provides valuable medium-term data, longer follow-up would be beneficial to assess the durability of these results beyond this timeframe.

Third, while we compared outcomes between the two ADM combination groups, propensity score matching was not performed. Although our multivariate analysis accounted for several key variables, potential unmeasured confounding factors might exist, warranting cautious interpretation of comparative results between the two techniques.

Furthermore, data on patient smoking status, a known factor potentially influencing tissue healing and integration, were not consistently available for analysis in our retrospective dataset, limiting our ability to assess its impact on ADM volume retention.

Finally, while aesthetic outcomes were assessed by a blinded panel, this study did not include formal patient-reported outcome measures (PROMs) using validated instruments like the BREAST-Q. Although the evaluation of aesthetic complications often incorporates patient concerns, our institution has now implemented the BREAST-Q Breast-Conserving Therapy module as a standard protocol for all oncoplastic procedures. A prospective study utilizing this validated instrument to systematically assess domains, including satisfaction with breasts, psychosocial well-being, physical well-being, and adverse effects of radiation, is currently underway and will provide comprehensive insights into the patient experience following ADM-based volume replacement.

Furthermore, data on patient smoking status, a known factor potentially influencing tissue healing and ADM integration, were attempted to be collected but were inconsistently documented in our retrospective dataset. Based on available records, approximately 18% of patients reported current or former smoking history, but this information could not be systematically analyzed across the entire cohort. In our current practice, we now prospectively document a detailed smoking history and recommend smoking cessation at least four weeks prior to surgery when ADM-based reconstruction is planned, recognizing its potential impact on wound healing and matrix incorporation.

Looking ahead, several areas warrant further investigation. Prospective studies incorporating standardized three-dimensional imaging would provide more precise volume measurements [38]. Investigation of patient-reported outcomes, particularly regarding satisfaction with breast symmetry and overall aesthetic results, would provide valuable insights into the patient experience [33]. Additionally, cost-effectiveness analyses comparing ADM-based volume replacement with traditional approaches would help inform resource allocation decisions [39,40].

## 5. Conclusions

Our study demonstrated that ADM provides predictable and durable volume replacement in oncoplastic breast-conserving surgery, with volume stability maintained even in the setting of radiotherapy. These findings have direct translational relevance for expanding breast conservation eligibility, potentially allowing surgeons to confidently offer breast-conserving options to patients with larger tumor-to-breast volume ratios, unfavorable tumor locations, or anticipated significant deformity who might otherwise be directed toward mastectomy. As the field of oncoplastic surgery continues to evolve, ADM-based volume replacement represents an important advancement in our ability to achieve optimal oncologic and aesthetic outcomes for breast cancer patients.

## Figures and Tables

**Table 1:** Patients’ baseline characteristics and operative data with between-group comparisons.

Variables	Total (n = 172)	Diced ADM and Sheet-Type ADM (n = 102)	Diced ADM and Paste-Type ADM (n = 70)	*p*-Value
**Reconstruction side**				0.68
Right	92 (53%)	56 (55%)	36 (51%)	
Left	80 (47%)	46 (45%)	34 (49%)	
**Age (years)**	49.3 ± 9.2	50.1 ± 8.7	48.1 ± 9.8	0.18
**BMI (kg/m^2^)**	23.7 ± 3.2	23.9 ± 3.1	23.4 ± 3.4	0.32
**Location**				0.41
Superomedial	48 (28%)	30 (29%)	18 (26%)	
Superolateral	72 (42%)	45 (44%)	27 (39%)	
Inferomedial	19 (11%)	9 (9%)	10 (14%)	
Inferolateral	33 (19%)	18 (18%)	15 (21%)	
**Radiotherapy**	170 (98.7%)	100 (98.0%)	70 (100%)	0.24
**Chemotherapy**	130 (75%)	79 (77%)	51 (73%)	0.49
Neoadjuvant chemotherapy	39 (23%)	22 (22%)	17 (24%)	0.68
Adjuvant chemotherapy	109 (63%)	65 (64%)	44 (63%)	0.92
**Histological type**				0.39
Invasive ductal carcinoma	123 (72%)	76 (75%)	47 (67%)	
Ductal carcinoma in situ	37 (22%)	19 (19%)	18 (26%)	
Invasive lobular carcinoma	6 (3%)	4 (4%)	2 (3%)	
Others	6 (3%)	3 (3%)	3 (4%)	
**Mean resected weight (g)**	21.5 ± 8.8	22.6 ± 7.9	20.3 ± 9.6	0.09
**Mean resected volume (cm^3^)**	20.9 ± 8.8	20.8 ± 7.8	21.1 ± 10.5	0.83
**Mean ADM volume (cm^3^)**	23.7 ± 6.2	25.2 ± 6.6	21.6 ± 4.8	<0.001

Values are expressed as mean ± standard deviation for continuous variables and as number (percentage) for categorical variables. Abbreviations: ADM, acellular dermal matrix; BMI, body mass index.

**Table 2:** Postoperative surgical complications.

Complications	Diced ADM and Sheet-Type ADM (n = 102)	Diced ADM and Paste-Type ADM (n = 70)	*p*-Value
Seroma	3 (2.9%)	1 (1.4%)	0.49
Hematoma	3 (2.9%)	4 (5.7%)	0.36
Infection	0	0	-
Flap necrosis	0	0	-
ADM non-incorporation	0	0	-
Total	6 (5.9%)	5 (7.1%)	0.74

**Table 3:** Comparison of postoperative ADM volumes measured by diameter-based and region-of-interest (ROI)-based methods at 6, 12, and 24 months.

Group	Postoperative Duration	Median Volume (cm^3^)		Volume Change (%)	*p*-Value
		Diameter-based	ROI-based	ROI-based	
Diced ADM and Sheet-type ADM (n = 102)	6 months	21.78 ± 8.53	23.21 ± 8.64	−0.4%	<0.001
	12 months	20.86 ± 8.16	22.50 ± 8.55	−3.1%	<0.001
	24 months	20.79 ± 8.19	22.22 ± 8.54	−4.3%	<0.001
Diced ADM and Paste-type ADM (n = 70)	6 months	18.35 ± 5.43	19.53 ± 5.97	−0.5%	<0.001
	12 months	17.91 ± 5.75	19.10 ± 5.98	−2.2%	<0.001
	24 months	17.80 ± 5.40	18.81 ± 5.96	−3.7%	<0.001

**Table 4:** Postoperative aesthetic complications.

Aesthetic Complications	Diced ADM and Sheet-Type ADM (n = 102)	Diced ADM and Paste-Type ADM (n = 70)	*p*-Value
Depression deformity	6 (5.8%)	5 (7.1%)	0.73
Bulging deformity	11 (10.7%)	6 (8.5%)	0.63
Dermal irregularity	0	2 (2.8%)	0.09
Total	17 (16.7%)	13 (18.6%)	0.74

## Data Availability

The data presented in this study are available upon request from the corresponding author. The data are not publicly available due to privacy restrictions and ethical considerations regarding patient information.
